# Phenotypic Variability and Genetic Diversity in a *Pinus koraiensis* Clonal Trial in Northeastern China

**DOI:** 10.3390/genes11060673

**Published:** 2020-06-19

**Authors:** David Kombi Kaviriri, Qinhui Zhang, Xinxin Zhang, Luping Jiang, Jinpeng Zhang, Jingyuan Wang, Damase P. Khasa, Xiangling You, Xiyang Zhao

**Affiliations:** 1 State Key Laboratory of Tree Genetics and Breeding, Northeast Forestry University, Harbin 150040, China; davidkaviriri@yahoo.fr (D.K.K.); zqh19950201@163.com (Q.Z.); zhangxinxin126@126.com (X.Z.); 15737928328@163.com (L.J.); yxiangling@yahoo.com (X.Y.); 2 Department of Natural Resources Management, Faculty of Agricultural Sciences, University of Kinshasa, P.O. Box 117, Kinshasa XI 023, Congo; 3 Department of Forestry, Faculty of Agricultural Sciences, Semuliki Official University, Beni 48, Congo; 4 Forest Cultivation Center, Linjiang Forestry Bureau of Jilin, Linjiang 134600, China; llzyckly@126.com (J.Z.); wjY7909@126.com (J.W.); 5 Canada Research Chair in Forest and Environmental Genomics, Centre for Forest Research and Institute for Integrative Biology and Systems, Université Laval, Quebec, QC GiV 0A6, Canada; damase.khasa@ibis.ulaval.ca

**Keywords:** clone classification, genetic diversity, *Pinus koraiensis*, phenotypic variation, microsatellite markers

## Abstract

Combining phenotypic and genetic characteristics in a genetic variation study is of paramount importance to effectively orient the selection of producers’ elite trees in a seed orchard. In total, 28 phenotypic characteristics and 16 microsatellite loci were used to analyze the clonal genetic variation, to characterize the genetic diversity, and to refine the genetic classifications of 110 *Pinus koraiensis* clones grown in the Naozhi orchard in northeastern China. All clones were significantly different in most traits. Most of the phenotypic characteristics showed great genetic variation among clones, while the genotypic differentiation was weak between the selection sites of clones. The SSR markers showed a relatively high level of genetic diversity (Na = 4.67 ± 0.43, Ne = 2.916 ± 0.18, I = 1.15 ± 0.07, Ho = 0.69 ± 0.04, He = 0.62 ± 0.02, and mean polymorphic information content (PIC) of 0.574), with higher heterozygosity as an indication of a lower probability of inbreeding events in the orchard. Despite weak correlation coefficients between dissimilarity matrices (r(A/B), range equal to 0.022, *p*-value < 0.001), the genetic and phenotypic classifications congruently subdivided all the clones into three major groups. The patterns of phenotypic trait variations and genetic diversity are valuable to effectively select materials in breeding programs of *P. koraiensis*.

## 1. Introduction

*Pinus koraiensis* Sieb. et Zucc (Korean pine) is an important economic timber species that dominates the mixed broadleaf-conifer forests in northeastern China [1]. Korean pine can reach a stem height of 30 m and a diameter of 150 cm at breast height [2]. *P. koraiensis* naturally occurs in a reduced area within northeastern China, north of North Korea, central Japan, and southeastern Russia [3]. It is widely appreciated for its use in internal and external construction, particularly due to the high quality of its wood [3]. The nuts of this species are also used for a variety of nutritional products and therapeutics [4]. Because of its various economic benefits, along with its increasingly recognized environmental roles in CO_2_ sequestration, water regulation and soil protection for plantations on slopes [5], the Korean pine has been extensively recommended for afforestation and reforestation projects [6]. Currently, artificial Korean pine forests are grown throughout northeastern Chinese provinces [7].

The *P. koraiensis* breeding program is considered among the oldest and largest forest tree improvement programs in China. Phenotypically superior Korean pine trees have been selected from natural forestlands and have been established in seed orchards since the early 1960s [8]. Many studies have evaluated the outcome of the Korean pine breeding program, and numerous elite tree lines have been selected based on phenotypic and genetic variability in growth, wood, fruit, seed and nutrient content within clonal, half-sib and full-sib *P. Koraiensis* populations [9,10]. Improved clones of *P. koraiensis* spp. were selected based only on their growth traits or wood properties to meet the demand for timber over the past several years [11]. However, with the official decree in 2016 against the cutting of natural forests, more attention has been paid to seeds and cones of *P. koraiensis* [4,12]. Considering only the seed yield, China is reported to have generated more than $250 million USD annually from pine nuts [13].

Although the selection of superior *P. koraiensis* trees and elite materials has traditionally been based on phenotypic characteristics, many genetic markers have been developed to characterize Korean pine genetic diversity [14,15] and assess clone fingerprints, paternity analysis, and pedigree reconstruction for breeding populations in tree improvement programs [16,17]. However, no study to date has linked the genetic variation in Korean pine with phenotypic characteristics and genetic markers.

Species genetic diversity highlights a pivotal role for trees under breeding programs to develop well-adapted tree varieties and heighten genetic gain for a multitude of useful traits [18,19]. To achieve genetic improvements, the breeder needs to maintain a certain amount of genetic diversity [20]. Maintaining high genetic diversity is a fundamental concern for breeders during recurrent selection operations. In fact, on one hand, breeders need to increase genetic gain by reducing the population size to only the elite individuals; on the other hand, breeders can increase the population’s adaptability through genetic diversity by guarding a large number of different genotypes [21,22]. 

The genetic diversity parameters measure the degree of resemblance and difference in the genetic characteristics of individuals or the population and could therefore guide assisted hybridization processes by preventing associations of genetically closed individuals [23]. Much research has shown a greater performance in the biomass, speed of development, and fertility in progeny compared to those of both parents defined as heterosis [24]. This has resulted from the heterozygosity (diversity) and dominance effect of crossed parents [25]. Reduced heterosis may result from the disharmonious functioning of alleles [26], but the heterosis effect will be greater when the crossed entities are genetically different [27].

The pairwise relatedness measurement displays a significant role in the genetic resource conservation. For instance, in a seed orchard, substantial effort would be made to ensure that close relative trees are not crossed to reduce inbreeding and reduce the loss of genetic variation by random genetic drift [28]. Using molecular markers, estimating the genetic relatedness between individuals can reveal correlations between genetic and phenotypic characteristics [29]. Microsatellite markers display high rates of polymorphism, good reproducibility, codominant inheritance and regular distribution in the coding and noncoding regions of the plant genome [30]. Because of that, microsatellites are recognized as the most efficient genetic markers for assessing genetic diversity and population genetic structure and for investigating relatedness between individuals [29]. 

The present study analyzed the phenotypic variability and genetic diversity of different clones (parent trees) and sought to establish their genetic similarities to lay the foundation for understanding vigor in offspring from possible combinations of different clone genotypes. In total, 28 phenotypic traits (11 growth traits; three wood traits; and 14 fruit, seed, and nut characteristics) were investigated and analyzed using 38-year-old *P. koraiensis* clones in the Naozhi seed orchard. The data of 16 codominant microsatellites were combined to compare genetic variation with phenotypic variability. Hence, the objectives of this study were to (i) analyze the clonal phenotypic and genetic variation, (ii) compare and combine phenotypic and genetic distances to assess the genetic diversity of *P. koraiensis*, and (iii) establish the genetic relationship among clones/genotypic relatedness based on phenotypic and molecular markers.

## 2. Materials and Methods 

### 2.1. Study Area and Plant Materials

Data were collected from the Naozhi forest seed orchard located on the western hillside of Changbai Mountain in Linjiang city, Jilin Province, northeastern China (41°05′ N, 126°06′ E). The *P. koraiensis* forest and broad-leaved mixed forest are typical forest vegetations of this region and occur at an altitudinal range between 700 m and 1100 m. The area experiences a continental monsoon climate type. In this region, the summer temperature is moderate because of the altitudinal gradient and rains, while the winter is long and cold. The average annual temperature and average annual rainfall range between 4 °C and 6 °C and 750 mm to 1000 mm, respectively. The frost-free period is 135 days. The soil type corresponds to the Albi-Boric Argo sols, according to U.S. soil taxonomy [31], and is dominated by dark brown soil of more than 40 cm thick, with a portion comprised of important textural constituents: sand (15.13%), silt (63.31%) and clay (21.56%) [32].

Tree measurements and plant materials were collected from surviving ramets of the 110 *P. koraiensis* clones (PK1-PK110). In 1979, superior Korean pine trees (based on their size) were selected from the natural *P. koraiensis* forestland in Linjiang, Jilin Province (41°55′33.86″ N, 127°01′33.26″ E) (PKJ-1 to PKJ-90) and Liaoning Caohe, Liaoning province (40°52′17.24″ N, 123°54′13.63″ E) (PKL-91 to PKL-110). These clones were grafted in the year following their collection, and plantations were established with four-year-old seedlings in the spring of 1984. The experimental design consisted of a complete randomised block design using single tree-plots, with 10 blocks containing 110 different clones, and ramets of each clone were planted at inter plant and row spacing of 7.0 m × 7.0 m. 

### 2.2. Growth, Wood, and Fruit Trait Measurements

The following traits were measured on living ramets in July 2018: 11 growth traits, including tree height (Ht), basal diameter (BD), diameter at breast height (DBH), diameter at 3-m height (DIAM-3m), bark thickness (BTH), stem volume (Vol.), stem straightness degree (SSD), branch angle (BA), crown breadth (CB), crown height (CH), and branch number (BNN); three wood traits, including wood density (WD), fiber length (FL), and fiber width (FWd); and 14 cone, seed, and nut traits comprising cone number (CN), cone length (CL), cone width (CWd), cone weight (CW), layer number (LN), seed number per cone (SNC), seed length (SL), seed width (SWd), seed weight (SW), nut length (NL), nut width (NWd), nut weight (NW), seed coat thickness (SCTH), and 1000 seed weight (1000 SW).

Ht and CH were measured with a meter ruler; BD, DBH, and DIAM3 were measured using a caliper; SSD was estimated and squared before calculation using the method of [33]. CB was calculated by averaging the values of the north-south and east-west crown width, and CH was obtained by subtracting the tree height at the first branch from the total tree height. The stem volume (Vol.) of each tree was computed according to the formula of Louppe [34] using a form coefficient of coniferous trees of 0.41, as shown in the following equation: $\mathrm{Vol}.={\mathrm{DBH}}^{2}\times \mathrm{Ht}\times \pi \times \mathrm{fc}/40000$, (π = 3.14 and fc = 0.41). 

From each clone, wood cores were collected from all ramets, following the altitudinal south-north orientation at breast height level (1.3 m) using an increment borer and were then taken to the laboratory for investigations of their wood properties. The WD and fiber dimensions (FL and FW) were measured according to the method used by Yin et al. [35].

For fruit-related traits, all the cones were collected from each ramet after reaching maturity. Cones were counted (CN) and directly weighed (CW). To obtain a balanced number of cones per clone, cones from previous years (2014 to 2018) were taken into account, and the calculated average was used in the analysis. Meanwhile, the cone size seeds and nuts were measured only in the year 2018. CL and CWd (perpendicularly at the broadest part) were measured with a digital caliper. After taking dimensions and weights from cones, all the seeds were removed from cones, and the number of seeds per cone per ramet was counted; the average value was used as cone SNC. To calculate the 1000 SW, seeds from different ramets were mixed. Then, 400 seeds were randomly chosen and divided into four equal parts to serve as replicates. Each group of 100 seeds was weighed, and the thousand seed weight was obtained by extrapolation from the weight of 100 seeds. Fifty seeds from each clone were randomly chosen to determine seed characteristics and measure SL, SWd, and SW. Finally, nuts were extracted from measured seeds, and NL, NWd and NW were assessed. SCTH was measured 10 times from different sides for each seed using a Vernier caliper, and the average values were used.

### 2.3. DNA Extraction and Simple Sequence Repeats (SSR) Analysis

Fresh needles were collected from each clone from an accessible branch. The collected needles were directly chilled under ice, transported to the laboratory and stored in a freezer at −80 °C for subsequent DNA analysis. Genomic DNA was extracted from *P. koraiensis* needles using a modified cetyltrimethylammonium bromide (CTAB) method, according to Allen et al. [36]. The DNA concentration and quality were tested using 1% agarose gel electrophoresis and a gel imaging system (Tanon-2500R, Tanon Science & Technology Co., Ltd., Shanghai, China). Sixteen published Simple Sequence Repeats (SSR) primer pairs were selected from previous studies ([16,37]) and used to perform PCR analysis (Table S1). 

DNA amplification (PCR reaction) was carried out in a total volume of 25 μL containing 2.5 μL of ExTaq buffer, 2 μL of dNTP (2.5 mM), 1 μL of MIX primer, 1 μL of plant DNA, 0.2 μL of ExTaq (Takara, Beijing, China), and 18.3 μL of H_2_O. The PCR was performed according to the following cycling program: denaturation step at 95 °C for 2 min; followed by 35 cycles of 95 °C for 20 s, annealing at 53~58 °C for 20 s (with different annealing temperatures for each primer pair), and 72 °C for 20 s for extension; and a final extension at 72 °C for 10 min. The reactions were then kept at 12 °C. All forward primer sequence markers were labeled with four fluorescent dyes: HEX (green), FAM (blue), TAMRA (yellow), and ROX (red). Amplified SSR fragments were analyzed using a capillary electrophoresis sequencer ABI 3730 XL DNA analyzer (Applied Biosystems, Foster City, CA, USA). The allele sizes were recorded using Gene Marker software V2.2.0 (Soft Genetics, State College, PA, USA). 

### 2.4. Statistical Analysis

Average clonal performances were evaluated, and the genotypic and environmental/random effects were estimated by the following linear mixed model, $Y_{\mathrm{ij}}=\mu +\beta_{i}+a_{\mathrm{ij}}+\varepsilon_{\mathrm{ij}}$ using R Software (Version 3.6.1) [38], with Yij as the performance of individual tree j of clone i, μ as the overall mean, $a_{\mathrm{ij}}$ as the random clonal value, $\beta_{i}$ as the fixed effect of block and $\varepsilon_{\mathrm{ij}}$ as the residual variance for each trait. Because each block contained a unique ramet from each clone, the interaction effect of clones and blocks was deleted from the model. The genotypic/clonal values were thus inferred through the relationship $a_{\mathrm{ij}}=Y_{\mathrm{ij}}-\mu -\beta_{i},$ and the variance components were calculated by $\sigma_{Yij}^{2}=\sigma_{ai}^{2}+\sigma_{\varepsilon }^{2},$ considering the microenvironmental conditions of blocks in the orchard to be identical. $\sigma_{ai}^{2}$, $\sigma_{\varepsilon }^{2}$ and $\sigma_{Yij}^{2}$ represented the genotypic variance, variance of within plot error, and total phenotypic variance, respectively [39]. Two-way analysis of variance (F test) was used to test the significance of growth differences in clones for all phenotypic traits. The clonal repeatability was estimated following [40] to determine the proportion of the total phenotypic variance that was due to the genotypes of clones by $H^{2}=\sigma_{ai}^{2}/\sigma_{Yij}^{2}$. Using clonal variances, the broad-sense genetic correlation was computed according to Liang et al. [9] by dividing trait covariance values by the square root of the sum of trait variances. The heat map package in R 3.6.1 was used to plot the genetic correlation using all the investigated phenotypic characteristics [41]. To measure the genetic divergence between the collections sites of the cloned genotypes, the formula proposed by Brommer, and Whither [42] was used as shown in the following relationship, $Q_{ST}=\sigma_{AB}^{2}/\sigma_{AB}^{2}+2\sigma_{AW}^{2}$, where $\sigma_{AB}^{2}$ and $\sigma_{AW}^{2}$ were the genotypic variances between and within clones from the selection site, respectively. 

Clonal phenotypic performances were standardized by subtracting the mean value and dividing by the standard deviation to construct an individual pairwise genetic distance using the Mahalanobis distance of dissimilarity. The dissimilarity matrix was then used to compare the genetic distances between phenotypic and molecular characteristics [43]. Finally, using clonal values, the clonal rank ordination of all the 110 cloned *P. koraiensis* genotypes was performed using an agglomerative hierarchical clustering (AHC) method [44]. The unweighted pair-group average method with the Pearson correlation coefficient as similarity indices was used to group all the clones that shared similar genotypic values from their phenotypic characteristics using XLSTAT software, (Boston, MA, USA. https://www.xlstst.com). [44]. 

Genotypic data of clones were recorded by SSR markers (allele sizes) using codominant values. First, clone kinship estimation using the SSR genotypes was done according to the protocol of Wu et al. [45]. The allele frequency analysis was performed based on the maximum likelihood method using CERVUS software version 3.0.7, (Field Genetics Ltd., London, U.K.), [46] and the range of allele frequency (Pi) and polymorphic information content (PIC) of each locus were computed [47]. Based on the plus tree origin selection sites, the genetic diversity parameters, including the effective number of alleles (Ne), number of different alleles per locus (Na), and Shannon’s Information Index (I), were computed according to [48], I = −1 × Sum (pi × Ln (pi)), with (pi) being the allele frequencies, and the observed heterozygosity (Ho), expected heterozygosity (He), unbiased expected heterozygosity (uHe), and fixation index (F) were calculated using GenAlEx 6.5.1b2, (Canberra, Australia, biology-assets.anu.edu.au/GenAlEx) [49]. The genetic differentiation (F_ST_) was retained from the analysis with GenAlEx software to test whether the two collection sites of clones were genetically different and to link genotypic differences to observed phenotypic characteristics. 

The Bayesian model of [50] integrated in STRUCTURE 2.3.4 software (Jonathan Pritchard lab, Stanford university, CA, USA) was used to analyze the genetic structure to verify the strength of a clone’s genetic relationship (clonal genetic grouping) using 10 independent runs and 5000 Markov Chain Monte Carlo (MCMC) repetitions after a burn-in period, as well as 5000 iterations for each group number of K that extended from 1 to 10. The method of utilizing the online tool Structure Harvester [51] and Structure Selector [52] were used to determine the likely number of genetic groups, and the optimum K value was obtained by the rate of change of the likelihood distribution (mean), as demonstrated by references.

A pairwise comparison of individual genetic distances was performed using Nei’s genetic distance method [53] in Power Marker software version 3.25 (Copyright © 2019, Informer Technologies, Inc., Los Angeles, CA, USA) [54]. The dissimilarity matrix was then used to construct a dendrogram using the Neighbor-joining method in Power Marker software. The interactive online tool “tree of life, ITOL” (www.itol.embl.de) [55] was used to visualize and edit the tree plot. Furthermore, an analysis of molecular variance (AMOVA) was conducted to determine the extent of the related individual genetic variation to the selected sites of the plus tree of different clones using GenALEx version 6.4.1 [49]. The comparison of the dissimilarity matrices (on phenotypic characteristics and SSR data) was performed using the mantel test in the XLSTAT 2 program 2019. 

## 3. Results

### 3.1. Phenotypic Variability

Clone variability and variation parameters in different traits were presented in Table 1. The phenotypic coefficient of variance for all characteristics ranged from 4.44% in stem straightness degree to 23.54% in stem volume. The proportion of variance that related to genotypes (clone repeatability) (Table 1) ranged from 0.02 in stem straightness degree to 0.98 in cone width. Traits related to the cone, seed, and nut were more stable, with repeatability values ranging from 0.80 for coat thickness to 0.98 for cone width, indicating that the observed differences in fruit characteristics were more related to clone identity than to the environment. These high repeatability values suggest that these traits could serve as an important basis for material selection. The average growth performances of clones was significantly different; furthermore, most of the characteristics showed moderate to very high levels of significant differences (0.05 ≤ *p*-value ≤ 0.001) based on ANOVA (Table 1), apart from stem straightness degree and cone number, for which the difference was not significant between clones, with *p*-values equal to 0.553 and 0.993, respectively. Highly significant differences were observed between blocks for some traits, indicating variation in environmental condition in the orchard. Collectively, these results demonstrate the feasibility of using the studied genotypes for classification purposes.

The differentiation analysis on the sites of collection based on the morphological characteristics show that the clones in the two collection sites were slightly different, with a phenotypic differentiation coefficient between 2 × 10^−6^ and 0.1901 for most of the morphological traits. However, some characteristics of the cones and seeds showed moderate to high differentiation coefficients, ranging between 0.4 and 0.9 (Table 1).

### 3.2. Classification Analysis of Phenotypic Characteristics

**Figure 1:**
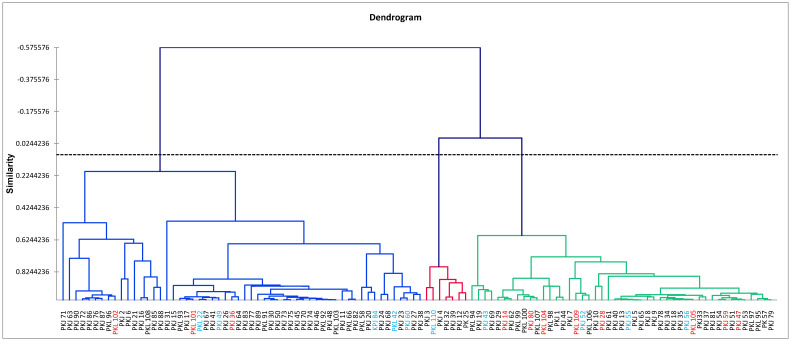
Clustering dendrogram showing cluster patterns of 110 *Pinus koraiensis* clones grown at the Naozhi orchard using the unweighted pair-group average method with Pearson correlation coefficient of similarity indices on 28 growth, wood, cone, seed, and nut traits. Genotypes in red and blue represent 10% of clones having exhibited high clonal value in volume and seed weight, respectively.

Cluster analysis was carried out on phenotypic traits in order to establish clonal ordination based on their performance (Figure 1) and to show the genotypic grouping (Figure S1). The clonal values (genotypic) from each trait were calculated and used to compute the similarity/dissimilarity distance indices from which an AHC method was used to construct a dendrogram with the dissimilarity matrix (Figure 1). Along the axis of individual distribution on the cluster dendrogram, three large groups of clones with similar phenotypic characteristics were detected from the 110 clones containing at least two subgroups. From left to right, the first cluster contained 56 clones, the second contained only seven clones, and the third contained 47 clones. Considering the origin of clones (selection site), all groups contained clones from both sites, showing no phenotypic specificity related to the origin selection site of the clones from the Naozhi forest and the Liaoning Caohe forestland.

**Figure 2:**
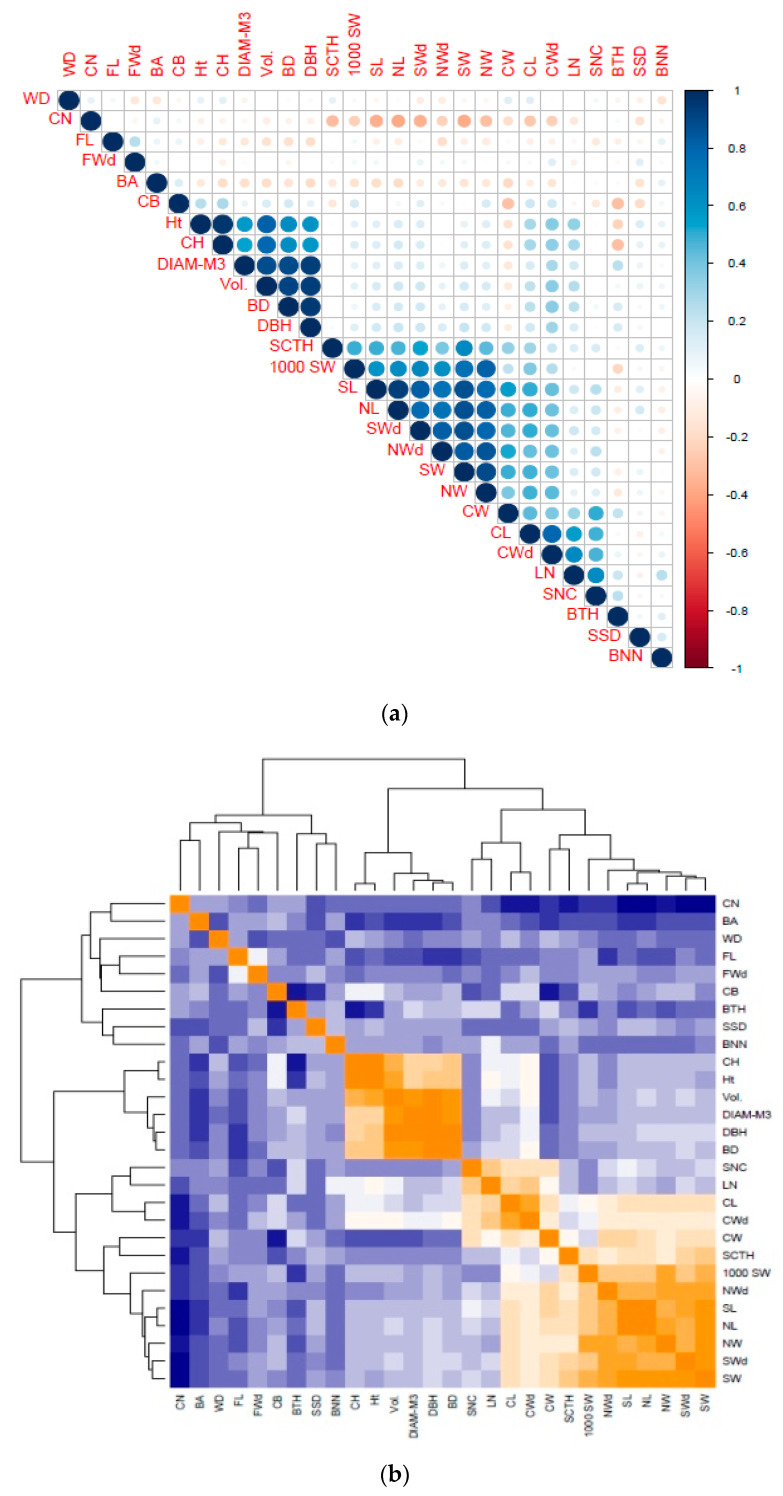
Correlation analysis of 28 growth, wood, cone, seed, and nut traits of 110 *P. koraiensis* clones grown at the Naozhi orchard. (**a**) Significance of correlation levels between traits and (**b**) clustering dendrogram of the correlated traits. WD: wood density; CN: cone number; FL: fiber length; FWd: fiber width; BA: branch angle; CB: crown breadth; Ht: tree height; CH: crown height; DIAM-M3: diameter at 3-m height; BD: basal diameter; DBH: diameter at breast height; SCTH: coat thickness; 1000 SW:1000 seed weight; CW: cone weight; SL:seed length; NL: nut length; SWd: seed width; NWd: nut width; NW: nut weight; SW: seed weight; CW: cone weight; CL: cone length; CWd: cone width; LN: layer number; SNC: seed number per cone; BTH: bark thickness; SSD: stem straightness degree; BNN: branch number per node.

The heatmap of correlation also showed three distinct clusters of traits in all the investigated phenotypic characteristics (Figure 2). From left to right, the first cluster was characterized by crown and wood traits; the second cluster by stem traits; and the third by cone-, seed-, and nut-related characteristics.

### 3.3. Clone Structure and Genetic Diversity

Sixteen SSR primers were used to analyze the 110 Korean pine clones from the Naozhi orchard. The SSR polymorphism data are given in Table S1 of the supplemental material. The lowest allele frequency range (Pi) was 0.005, and the highest was 0.764. The average polymorphic information content (PIC) of the 16 SSR loci was 0.574 (Table 2). The genetic variation parameters of clones are presented in Table 1. The mean number of alleles per locus (Na) was 4.67 ± 0.43 and ranged from 2.00 ± 0.00 to 9.00 ± 2.00. The average number of effective alleles (Ne) was 2.916 ± 0.18 and ranged from 1.53 ± 0.06 to 4.35 ± 0.42. The mean unbiased expected heterozygosity (uHe, 0.63 ± 0.02) and the mean expected heterozygosity (He, 0.62 ± 0.02) were lower than the mean observed heterozygosity (Ho, 0.69 ± 0.04), with a fixation index (F) of −0.11 ± 0.03. The calculated Shannon’s Information Index (I) was 1.15 ± 0.07. The analysis of molecular variance (AMOVA) revealed that the primary source of genetic variance (96%) was within the collection sites of clones, and a small amount of variation (4%) appeared between the selection sites of clones (Table 3), indicating that there were allele exchanges by genetic flux between the two sites. A weak genetic differentiation among the site of collection (0.03 ± 0.01) was also indicated by pairwise Fst ranging from 0.001 to 0.077 (Table 2).

### 3.4. Cluster Analysis by Molecular Markers

**Figure 3:**
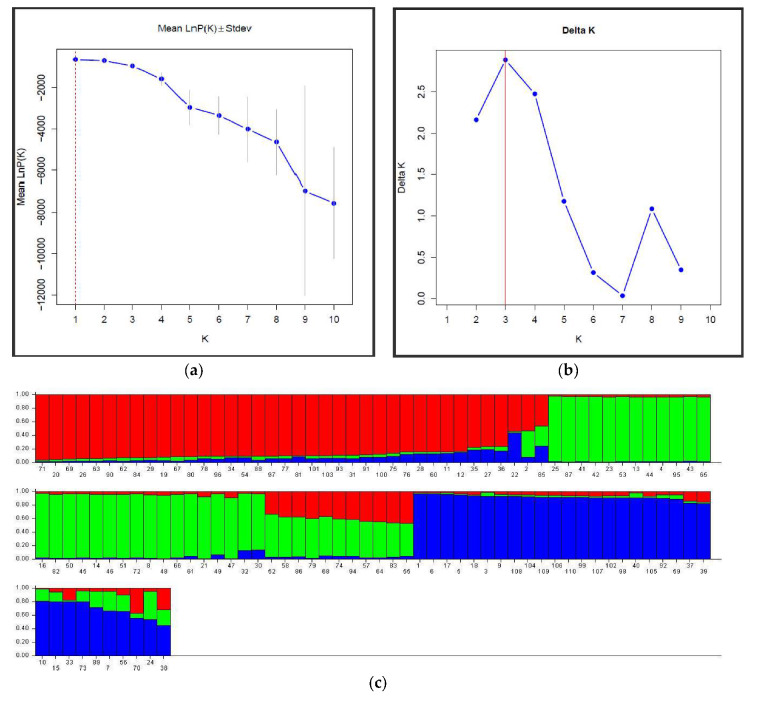

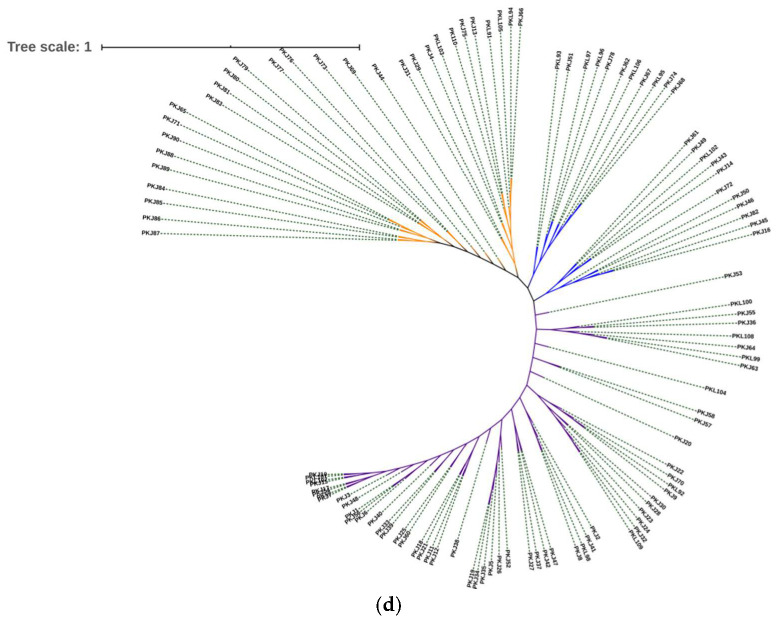
Genetic structural and cluster analyses of 110 *P. koraiensis* clones. (**a**) The probability of the data ln P(D) (+SD) against the number of K clusters; (**b**) ΔK values from the mean log-likelihood probabilities inferred clusters (K); (**c**) Estimated genetic clustering (k = 3) and (**d**) showed clusters based on Nei’s genetic distance. The *x*-axis in (**c**) indicates the clone’s number, and the *y*-axis value shows group membership.

Genetic structure analysis was performed using STRUCTURE software to check the strength of genetic grouping of the 110 *P. koraiensis* clone genotypes. The number of subgroupings was not clearly identified from the plot of Ln (probability of data) for K (Figure 3a). However, using the ΔK method, three clusters were revealed, corresponding to the highest ΔK value found, K = 3 (Figure 3b). A few individual admixtures were observed between clusters, indicating weak differentiation (Figure 3c). The 110 Korean pine genotypes were divided into three clusters (Figure 3c)—Cluster 1 (red) contained 38 individuals, Cluster 2 (green) included 40 individuals, and Cluster 3 (blue) included 32 clone genotypes. Three major groups were also observed on the neighbor-joining clustering dendrogram (Figure 3d) based on Nei’s genetic distances on the SSR data. All the clones were mixed in the formed groups, confirming the genetic flux between natural lands of Korean pine. 

### 3.5. Correlation between Phenotypic and Molecular Markers

**Figure 4:**
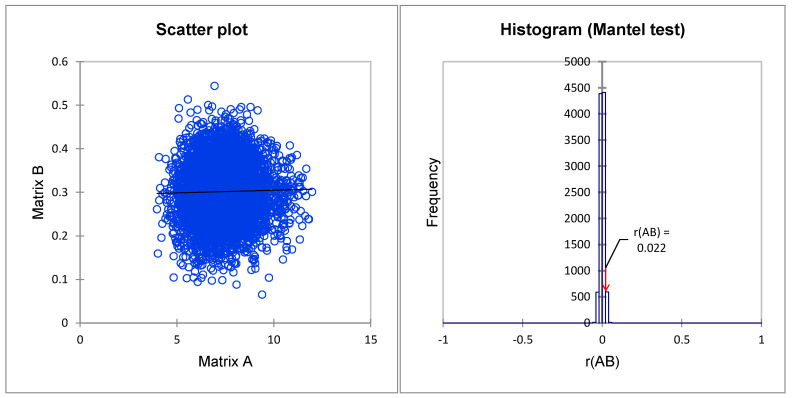
Correlation analysis between dissimilarity matrices of clonal genetic relatedness from 16 SSR markers and clonal genotypic values of 28 phenotypic traits.

To determine the correspondence level between the dissimilarity matrices based on phenotypic characteristics and SSR markers, a Mantel test of correlation was performed. The correlation result is shown in Figure 4. There was a significant positive correlation between the dissimilarity matrices on the phenotypic and genetic distances r(A/B) = 0.022, (α = 0.05, *p*-value < 0.001), suggesting that the molecular and phenotypic categorizations of *P. koraiensis* were nearly identical.

## 4. Discussion

### 4.1. Clone Variability Based on Phenotypic Characteristics

In general terms, phenotypic characteristics are considered the basic elements for detecting dissimilarities/similarities between individuals from the same or different tree species [56]. In tree breeding research, growth characteristics (i.e., stem height and diameter) are widely used to select various varieties according to different growing sites in several tree improvement programs [57]. Wood properties, such as fiber dimensions (length and width) and fruit characteristics, are associated with growth traits to assess whether the scope of the tree affects the quality of wood or produced paper [58] and also to determine if the fruit quantity and/or quality are dictated by tree growth size [59]. The present study combined phenotypic and genetic characteristics to assess phenotypic variation and genetic diversity. Preliminary studies showed high phenotypic variation in multiple traits. To highlight the intersection between phenotypic variability and genetic diversity, 28 phenotypic characteristics (11 stem and 17 fruit traits), and 16 microsatellite markers were used in this study.

The mean growth performances showed a significant difference among clones for most growth traits, indicating the effects of clone genotype were significant, and this result was supported by a clonal repeatability greater than 70% for these characteristics. This result is promising for the selection of *P. koraiensis* clones. Similar findings were reported on many tree species, indicating that selection based on phenotypic variation results is feasible [60,61,62,63]. Considering the extent of phenotypic variation, the clone variability in PCV values (%) seems to be slightly lower compared to prior *P. koraiensis* variation studies [9,10]; this is likely due to the age difference in Korean pine materials used in each study [61], the genetic properties (clone against families), planting design and different environmental conditions. The material age is an important factor in growth variation of planted trees that share similar environmental conditions, and growth sizes seem to be uniform among mature trees [64]. 

The genetic variation parameters evaluated in this work on phenotypic traits seem to be higher, especially in fruit-related traits, with repeatability values up to 90%. A previous study of [65] reported heritability values of less than 0.7 in fruit characteristics while investigating 33 years of Korean pine half-sib families. The variation in environmental conditions in the orchard would have influenced the variation, given significant growth differences from subblocks obtained in several growth characteristics. Indeed, with unique ramets for each clone in the subblocks, the effect of the interaction of clones and blocks in the growth model was not taken into account. This could have overestimated the performance of the clones. However, these results retained their value based on the overall assessment of the growth of clones on the whole seed orchard. 

### 4.2. Cluster Analysis Based on Phenotypic Traits

All 110 clones exhibited a strong significant difference in most of the investigated traits. However, the clustering dendrogram (Figure 1) showed three large distinct groups of clones, displaying different high-performance values in different growth traits. Taking into account the trait correlations, three different clusters of characteristic traits were also detected from the clustering dendrogram built according to phenotypes (Figure 2). Since these trees were planted in a randomized design with more or less identical soil and climatic conditions, the differences in growth and phenotypic traits between clones might be related to the genetic properties of the clones [66]. Cluster analysis has been widely used in the classification of plant genetic material. Dendrograms were constructed using morpho-physiological traits for *Prunus avium* L. [67] and *Festuca arundinacea* Schreb. [68], along with morphological traits for *Medicago sativa* L. [68], suggesting that useful information can be obtained from the classification of plant materials. 

### 4.3. SSR Polymorphism and Clone Genetic Diversity

Since phenotypic markers are affected by the environment, they are less useful for characterizing neutral genetic diversity to effectively capture the genetic diversity required to maintain a wide genetic base in future breeding programs [69,70,71,72,73]. The use of genetic markers overcame this selection error mostly because genetic markers are not affected by the environment and can be detected at any development stage of the tree [74]. SSR marker techniques have been widely used to detect genetic variation and estimate population genetic diversity and individual relatedness levels in many tree populations. The 110 *P. koraiensis* clones in this study showed high genetic diversity based on SSR marker analysis. The average number of alleles and effective alleles per locus (Na, 4.67 ± 0.43; Ne, 2.916 ± 0.18) and the mean He (0.62 ± 0.02) and uHe (0.63 ± 0.02) were higher compared to the diversity parameters reported for *Pinus thunbergii* [75]. However, the values determined in this study were higher compared to results reported by [76,77], which analyzed the mating system in a complete *P. koraiensis* population containing seeds, male and female trees using 14 SSR markers. Our results corroborated previous studies [78,79], which indicated higher genetic diversity in Chinese natural Korean pine populations compared to that of other populations in the Russian region. 

The PIC, Shannon’s Information Index (I), and Fixation Index (F) were 0.5736 ± 0.294, 1.15 ± 0.07, and −0.11 ± 0.03, respectively, and were comparable to the findings of [16] that analyzed SSR markers. The great genetic diversity observed in *P. koraiensis* is an essential factor for improving the efficient selection of genotypes that might resist different environmental hazards (climate, parasites, etc.) [80]. Several factors, including the mating system, affect the maintenance of high genetic diversity in tree populations to varying degrees [81,82,83]. One study [16] demonstrated that the pollen source of *P. koraiensis* was greatly affected by wind direction during the pollination season. The Fixation Index (F) of −0.11 ± 0.03 that we found in this orchard complex suggested strong inbreeding. Similar results of limited heterozygotes in seed orchard materials have been reported by other workers (e.g., Arnaud-Haond et al. [83]), while other authors have reported a slight excess of heterozygosity (e.g., Nybom [84]).

### 4.4. Genetic Structure and Clone Genetic Relatedness 

The genetic structure of tree species is affected by numerous factors, including population size, mating systems, genetic drift, gene flow, seed dispersal, natural selection, and evolutionary history [19,85]. The impact of a change in genetic structure can be serious, especially when it affects the mating system or the process of gene flow. Any planting design in a seed orchard is a change to the natural genetic structure and may bring together genetically closed or genetically distant individuals, thus affecting the structure of future generations [48,86]. Our analysis of genetic structure showed that the 110 clones formed three genetic groups with a few admixed individuals, indicating weak genetic divergence among clones from the two clone collection sites (Figure 3c). AMOVA results also revealed that only 4% of variation was accounted for among collection sites, while 96% of genetic variation occurred among clone/within clone collection sites (*P* < 0.001) (Table 3). This finding may be explained by the fact that the two collection sites are located in the southwest region of a large distribution area of Korean pine and benefit from similar propagation conditions. The two sites are 476 km apart, which appears to be wide enough to prevent an effective gene flow [87] for this wind-pollinated species [69,88]. The comparison of both hierarchical-joining cluster results (Figure 1), the Bayesian STRUCTURE (K = 3) bar plot (Figure 3c), and neighbor-joining cluster results (Figure 3d) showed a relative approximation in the interpretation between the genetic relationships and the clone classification dendrogram. A similar observation was previously made [49] in a study that found a parallel trend from the classification of a *Salix psammophila* populations in both clustering analysis methods. 

### 4.5. Correlations between Phenotypic and Molecular Markers 

The Mantel test [89], which calculates the association between two distance matrices, is widely used to test the linear or monotonic independence of subjects in two distance matrices [90]. As described previously, the Mantel approach is an appropriate test when the hypothesis is formulated in terms of distance matrices (as is the case with genetic data) [90]. In the present study, the Mantel test was used to compare the clone dissimilarity matrix based on phenotypic characteristics and SSR data. The results showed a weak correlation but a positive significant relationship between the distance matrices (Figure 4). This weak correlation is due to the nature of the data and different calculation approaches in distance matrices [91,92]. A low positive correlation was found between SSR data and morpho-physiological traits of tall fescue accessions [67]. In contrast, this study used the agglomerative hierarchical clustering method to identify clones with similar genotypes and phenotypic characteristics. Three groups were detected in both dendrograms (Figure 1 and Figure 3). The correlation in dissimilarity matrices of both classification methods in this study would result in a large number of investigated phenotypic traits (28 characteristics). Ref. [93] showed that a greater number of phenotypic markers could yield a better representation of genetic distances, especially in a small gene pool with a weak environmental effect [94]. 

## 5. Final Considerations

The present study focused on the variation in phenotypic traits and genetic relationships that exist among a large number of genotypes from clones from plus trees of *P. koraiensis*, which will be used to improve growth and seed production and to maintain a wide genetic diversity in future breeding programs. All the clones were phenotypically different at moderate to high levels of significance. The phenotypic difference was stronger due to the genetic properties of clones than to the environment and physiological effects. A significant relationship was observed between clone classification based on the phenotypic and genetic distances matrices. The results based on the SSR techniques revealed a high level of genetic diversity in the *P. koraiensis* clones, which is essential for effective selection regarding the reduced and nearest area in the collection sites and the number of individuals in this study. Three main groups were identified by both genetic and phenotypic data.

Based on this work, growth genotypic values and genetic characteristics of 110 *P. koraiensis* cloned trees at the Naozhi orchard were determined. The classification dendrogram allows identification of whether selectable clones display similar performances in terms of genotypic/clonal values on the ordination axis that may be done on growth performance-based volume and seed weight. For instance, a 10% slice of high performing materials, which later would serve as selection objects for wood products or seed qualities, showed relatively low to high similarity coefficients ranging between 0.22 and 0.82. The different genotype groups allow one to determine whether these potentially selectable materials are genetically near or distant from each other in order to prevent inbreeding, which could impact the genetic properties of seeds that are produced and the long-term future improvement of the species.

## Figures and Tables

**Table 1:** Descriptive statistics and variation parameters of 110 *Pinus koraiensis* clones grown in the Naozhi seed orchard.

Traits	Units	SV	SS	df	MS	F	PCV	H^2^	*Q_ST_*
Tree height	(m)	Clones	620.899	109	5.696	2.965 ***	8.36	0.663	0.0236
		Blocks	89.767	9	9.974	5.191 ***			
Basal diameter	(cm)	Clones	7287.222	109	66.855	3.981 ***	9.05	0.749	0.0761
		Blocks	384.485	9	42.721	2.544 **			
Diameter at breast height	(cm)	Clones	6810.669	109	62.483	4.260 ***	9.3	0.765	0.0036
		Blocks	118.065	9	13.118	0.894 ^***^			
Diameter at 3-m height	(cm)	Clones	6228.944	109	57.146	3.685 ***	9.55	0.729	0.0258
		Blocks	162.950	9	18.106	1.167 *			
Stem volume	(m3)	Clones	6.985	109	0.064	4.490 ***	23.54	0.777	0.0003
		Blocks	0.254	9	0.028	1.976 **			
Burk thickness	(mm)	Clones	542.443	109	4.977	1.976 ***	12.2	0.494	0.0122
		Blocks	159.142	9	17.682	7.020 ***			
Stem straightness degree		Clones	5.469	109	109	2.022 ***	4.44	0.505	0.0001
		Blocks	8.701	9	0.967	38.963 ***			
Branch angle	(°)	Clones	114,639.207	109	1051.736	2.063 ***	9.18	0.515	0.6407
		Blocks	3209.167	9	356.574	0.699^N^			
Crown breath	(m)	Clones	77.354	109	0.710	2.168 ***	8.03	0.539	0.0003
		Blocks	46.356	9	5.151	15.738 ***			
Crown height	(m)	Clones	601.50	109	5.52	2.72 ***	10.69	0.633	0.0076
		Blocks	52.15	9	5.79	2.86 ***			
Branch number per node		Clones	94.641	109	109	1.455 **	13.01	0.313	0.0034
		Blocks	12.895	9	1.433	2.400 *			
Wood density	(g/cm^3^)	Clones	1.025	109	0.009	1.991 ***	13.20	0.498	2.00 × 10^−5^
		Blocks	0.016	9	0.008	1.644^N^			
Fiber length	(µm)	Clones	281,044,500.967	109	2,578,389.917	4.664 ***	11.13	0.786	0.0992
		Blocks	2,491,085,275.474	9	85,899,492.258	155.372 ***			
Fiber width	(µm)	Clones	12,701.725	109	116.530	2.324 ***	5.71	0.570	0.1901
		Blocks	57,119.958	9	1969.654	39.277 ***			
Cone number		Clones	925.100	109	109	0.686^N^	17.98	0.457	0.0183
		Blocks	60.502	9	6.722	0.539^N^			
Cone length	(mm)	Clones	27,1671.646	109	2492.400	13.650 ***	8.91	0.927	0.0869
		Blocks	6518.537	9	210.275	1.152 *			
Cone width	(mm)	Clones	16,5848.289	109	1521.544	43.426 ***	13.25	0.977	0.4178
		Blocks	1231.719	9	39.733	1.134 *			
Cone weight	(g)	Clones	2,370,353.219	109	21,746.360	10.751 ***	16.04	0.907	0.9809
		Blocks	53,424.308	9	1723.365	0.852^N^			
Layer number		Clones	4149.593	109	109	10.607 ***	11.16	0.906	0.2264
		Blocks	208.822	9	6.736	1.877 ***			
Seed number per cone		Clones	352,729.157	109	109	5.892 ***	9.73	0.830	0.4171
		Blocks	46,422.283	9	1497.493	2.727 ***			
Seed length	(mm)	Clones	1510.20	109	13.86	10.87 ***	5.86	0.908	0.0388
		Blocks	191.65	9	13.69	10.74 ***			
Seed width	(mm)	Clones	739.32	109	6.78	5.84 ***	6.28	0.829	0.0059
		Blocks	96.10	9	6.86	5.91 ***			
Seed weight	(g)	Clones	20.32	109	0.19	16.37 ***	15.66	0.939	0.0002
		Blocks	2.40	9	0.17	15.07 ***			
Nut length	(mm)	Clones	1242.77	109	11.40	12.22 ***	6.51	0.918	0.0188
		Blocks	126.41	9	9.03	9.68 ***			
Nut width	(mm)	Clones	279.18	109	2.56	5.62 ***	5.84	0.822	0.0004
		Blocks	31.92	9	2.28	5.01 ***			
Nut weight	(g)	Clones	3.55	109	0.03	6.72 ***	17.17	0.851	3.00 × 10^−5^
		Blocks	0.44	9	0.03	6.50 ***			
Coat thickness	(mm)	Clones	6.65	109	0.06	5.04 ***	6.97	0.801	2.00 × 10^−6^
		Blocks	0.51	9	0.04	3.03 ***			
1000 seed weight	(g)	Clones	3,082,247.35	109	28,277.50	11.47 ***	13.37	0.913	0.9958
		Blocks	6090.76	9	1015.13	0.41^N^			

Note: SV, SS, df, MS, PCV, F, H^2^ and *Q**_ST_*** represent the source of variance, sum square, degrees of freedom, mean square, phenotypic coefficient of variance, F Fisher-Yates coefficients, repeatability and between site genetic divergence respectively. *** represents a highly significant difference (*p*-value ≤ 0.001), ** represents a significant difference (*p*-value ≤ 0.05), * represents a significant difference (*p*-value ≤ 0.05) and NS indicates no significant difference.

**Table 2:** Diversity statistics of the 16 microsatellite markers on 110 *P. koraiensis* clones grown at the Naozhi seed orchard.

Locus	Na	Ne	I	Ho	He	uHe	F	Fst
PCP45071	7.00 ± 0.00	4.10 ± 0.88	1.59 ± 0.11	0.92 ± 0.02	0.75 ± 0.05	0.76 ± 0.05	−0.24 ± 0.07	0.042
Pt79951	3.00 ± 1.00	2.02 ± 0.09	0.75 ± 0.08	0.58 ± 0.02	0.51 ± 0.02	0.51 ± 0.02	−0.16 ± 0.09	0.003
10F/RR	8.00 ± 0.00	4.35 ± 0.42	1.66 ± 0.08	0.79 ± 0.11	0.77 ± 0.02	0.78 ± 0.03	−0.03 ± 0.12	0.053
P11	3.00 ± 1.00	1.98 ± 0.12	0.77 ± 0.11	0.55 ± 0.03	0.49 ± 0.03	0.50 ± 0.02	−0.11 ± 0.07	0.002
P25	2.00 ± 0.00	1.98 ± 0.09	0.69 ± 0.01	0.55 ± 0.03	0.49 ± 0.01	0.50 ± 0.01	−0.11 ± 0.01	0.002
P44	3.50 ± 0.50	2.26 ± 0.24	0.96 ± 0.02	0.66 ± 0.14	0.55 ± 0.05	0.56 ± 0.05	−0.19 ± 0.15	0.034
P49	4.00 ± 0.00	3.23 ± 0.28	1.26 ± 0.06	0.71 ± 0.04	0.69 ± 0.03	0.69 ± 0.03	−0.04 ± 0.01	0.034
P60	9.00 ± 1.00	4.37 ± 0.45	1.75 ± 0.06	0.92 ± 0.03	0.77 ± 0.02	0.78 ± 0.02	−0.20 ± 0.08	0.048
P60	9.00 ± 2.00	4.22 ± 0.15	1.69 ± 0.01	0.94 ± 0.04	0.76 ± 0.01	0.78 ± 0.02	−0.24 ± 0.07	0.001
P62	3.00 ± 0.00	2.20 ± 0.03	0.90 ± 0.01	0.60 ± 0.10	0.55 ± 0.01	0.55 ± 0.01	−0.10 ± 0.20	0.020
P63	5.00 ± 0.00	3.25 ± 0.55	1.32 ± 0.12	0.81 ± 0.09	0.68 ± 0.05	0.69 ± 0.06	−0.19 ± 0.04	0.029
P79	5.00 ± 1.00	2.97 ± 0.64	1.24 ± 0.25	0.81 ± 0.06	0.65 ± 0.07	0.66 ± 0.07	−0.27 ± 0.05	0.003
P74	2.00 ± 0.00	1.53 ± 0.06	0.53 ± 0.03	0.41 ± 0.01	0.35 ± 0.03	0.35 ± 0.02	−0.20 ± 0.05	0.024
P82	4.00 ± 1.00	3.19 ± 0.78	1.29 ± 0.24	0.69 ± 0.04	0.67 ± 0.08	0.68 ± 0.08	−0.04 ± 0.07	0.077
P90	4.00 ± 0.00	2.47 ± 0.20	1.11 ± 0.05	0.56 ± 0.11	0.59 ± 0.03	0.60 ± 0.03	0.06 ± 0.13	0.003
P92	3.00 ± 0.00	2.55 ± 0.27	0.99 ± 0.07	0.43 ± 0.23	0.60 ± 0.04	0.61 ± 0.05	0.26 ± 0.43	0.044
Mean	4.67 ± 0.43	2.916 ± 0.18	1.15 ± 0.07	0.69 ± 0.04	0.62 ± 0.02	0.63 ± 0.02	−0.11 ± 0.03	0.03 ± 0.01

Note: Na = No. of alleles per locus; Ne = No. of effective alleles = 1/(Sum pi^2^), I = Shannon’s Information Index = −1 * Sum (pi * Ln (pi)), Ho = Observed Heterozygosity = No. of Hets/N, He = Expected Heterozygosity = 1 − Sum pi^2^, uHe = Unbiased Expected Heterozygosity = (2N/(2N − 1)) * He, F = Fixation Index = (He − Ho)/He = 1 − (Ho/He), Fst = (Ht − Mean He)/Ht and Ht = Total Expected Heterozygosity = 1 − Sum tpi^2^, where tpi is the frequency of the ith allele for the total, and Sum tpi^2^ is the sum of the squared total allele frequencies.

**Table 3:** Analysis of molecular variance (AMOVA) of 110 *P. koraiensis* clones grown at the Naozhi seed orchard.

Source	df	SS	MS	Est. Var.	%
Among collection sites	1	19.680	19.680	0.224	4%
Within collection sites	218	1094.361	5.020	5.020	96%
Total	219	1114.041	−	5.244	100%

## Supplementary Materials

The following are available online at https://www.mdpi.com/2073-4425/11/6/673/s1, Figure S1: Clustering dendrogram showing cluster patterns of 110 *Punis koraiensis* clones grown at the Naozhi orchard using the unweighted pair-group average method with Mahalonobis coefficient of disimilarity indices on 28 growth, wood, cone, seed and nut traits., Figure S2: Genetic structural and clusters grouping of 110 *Punis koraiensis* clones based on SSR data using Structure Selector, Table S1: Primer sequences and polymorphism characteristics of SSR markers used to characterize 110 *P. koraiensis* clones grown at the Naozhi seed orchard.
