# Supplementary material for: Phenotypic Variability and Genetic Diversity in a Pinus koraiensis Clonal Trial in Northeastern China
Source: Genes (Basel). 2020 Jun 19;11(6):673. doi: 10.3390/genes11060673 (PMC7348814; doi:10.3390/genes11060673)
Supplement: Supplementary file 1 [file genes-11-00673-s001.pdf]

Supplementary Materials

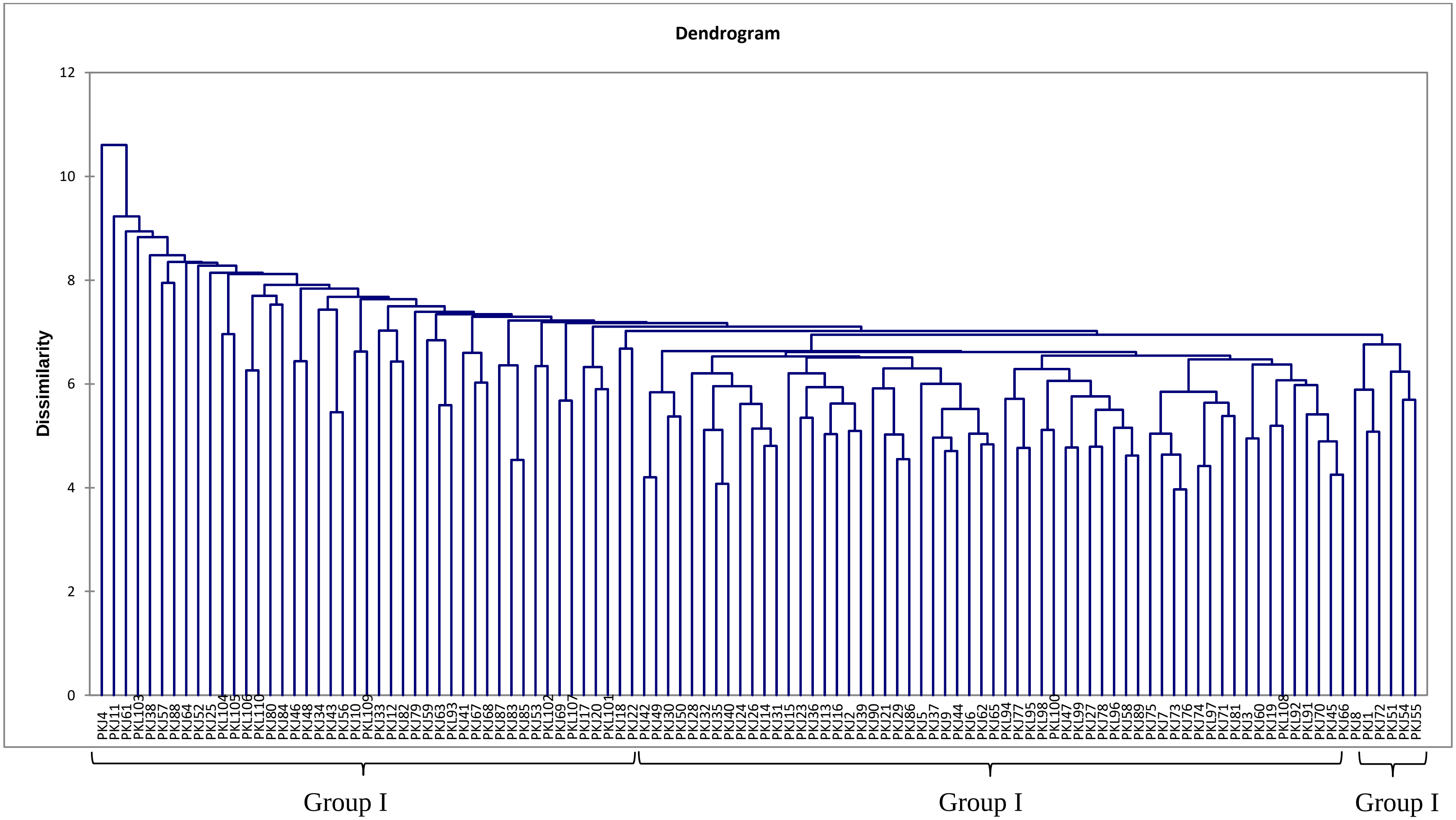

Figure, S1. Clustering dendrogram showing cluster patterns of 110 *Punis koraiensis* clones grown at the Naozhi orchard using the unweighted pair-group average method with Mahalonobis coefficient of dissimilarity indices on 28 growth, wood, cone, seed and nut traits.

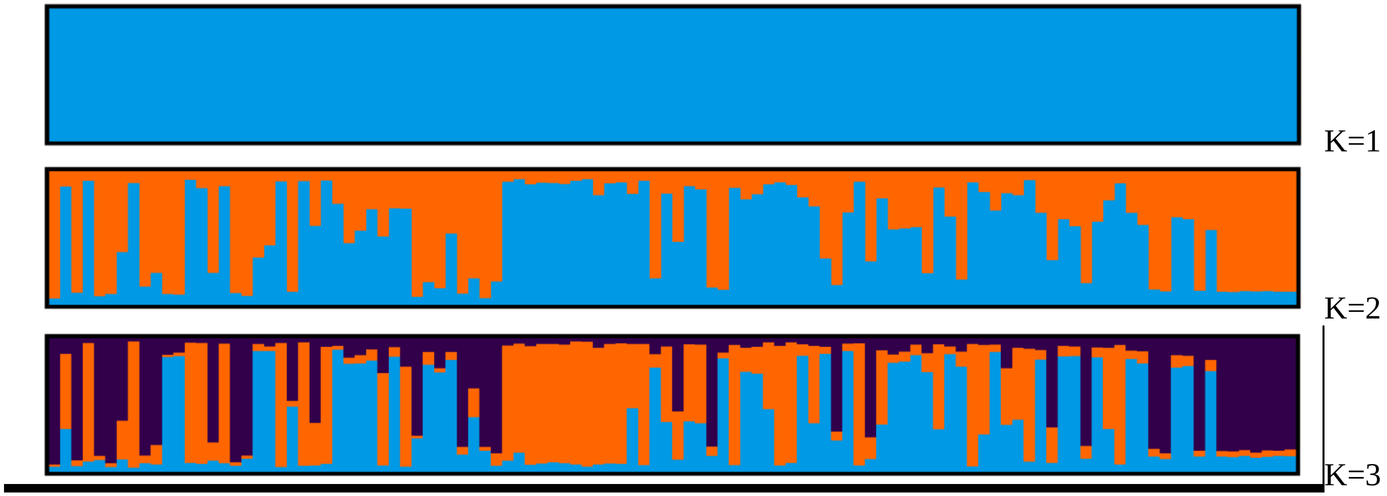

Figure, S2. Genetic structural and clusters grouping of 110 *P. koraiensis* clones based on SSR data using Structure Selector, [51]

Table S1. Primer sequences and polymorphism characteristics of SSR markers used to characterize 110 *P. koraiensis* clones grown at the Naozhi seed orchard.

Locus	(F-S) Primer sequences (5′-3′)	Allele size range (bp)	Allele frequency Range (Pi)	Polymorphic information content (PIC)
PCP45071	ACTGGTCTGATCGACCCAAT<br>TTCTACACTTGCGGAAACCC	150-167	0.005-0.346	0.770
Pt79951	CTTTTGTTTTTCAACAATTGCA<br>ACATCTATCTCCCATATCGGC	147-154	0.009-0.036	0.410
10F/RR	CAGAAGCCCAAGCTTATGGC<br>CGGATTGATCCTAACCATAC	201-216	0.005-0.550	0.749
P11	TGAGAATGAGGCGAACTG<br>GAAGGAAAGGTAAGGTGGA	314-331	0.009-0.605	0.424
P25	AAAGTTCACATTGGCACATC<br>TCAGTCCAGCGACAACAG	455-459	0.009-0.309	0.371
P44	TTTCGGTTCTCAGGCTCT<br>CCCTGGTGGTACAATGAC	182-195	0.009-0.259	0.478
P49	GAGATGAGCGAATCTGGG<br>TACAAGTTCCACCTACGG	255-262	0.027-0.632	0.478
P60	AAACGCAGAGTGGAGGAA<br>AACTCGGAGCATTTGGTG	234-251	0.105-0.632	0.771
P62	AGTGGTCTACGCTGGAGT<br>AACATTTAGGTCTTGGAGG	129-133	0.041-0.491	0.469
P63	GCAGCAGATCAGAGGGAG<br>CAGCCAACAACTGGTCATAC	132-155	0.082-0.436	0.605
P67	TGAACGCACAGGCAAGTT<br>GCGAAGGCAATGGTGAAA	259-260	0.027-0.323	0.750
P74	ACGCTACCGATTCTTACC<br>GTGTTCGCCTACAACTCAT	135-140	0.091-0.573	0.467
P79	CCACCGCCAAGTCCATTA<br>GCTTTGTTAGCCGTCCAG	180-200	0.064-0.464	0.667
P82	GGAAGATGAATCGCAAACC<br>ACACCCGCCTGAAGAGCA	272-294	0.236-0.764	0.296
P90	CCGCAAATCCGAGCAATG<br>GCAGCAGACGATAATGAACCC	134-139	0.105-0.564	0.568
P92	ACTTTGCGTGAATCAGACC<br>AAAGTAAGGCTGCTTGCATGA	249-250	0.053-0.553	0.541
Mean	-	-	-	0.5736 ±0.294

$$\text{PIC} = 1 - \left(\sum PI\right)^2, [93]$$
